# TRIB1 Is Regulated Post-Transcriptionally by Proteasomal and Non-Proteasomal Pathways

**DOI:** 10.1371/journal.pone.0152346

**Published:** 2016-03-28

**Authors:** Sébastien Soubeyrand, Amy Martinuk, Paulina Lau, Ruth McPherson

**Affiliations:** 1 Atherogenomics Laboratory, University of Ottawa Heart Institute, Ottawa, Canada; 2 Division of Cardiology, University of Ottawa Heart Institute, Ottawa, Canada; CNRS UMR7275, FRANCE

## Abstract

The *TRIB1* gene has been associated with multiple malignancies, plasma triglycerides and coronary artery disease (CAD). Despite the clinical significance of this pseudo-kinase, there is little information on the regulation of TRIB1. Previous studies reported *TRIB1* mRNA to be unstable, hinting that TRIB1 might be subject to post-transcriptional regulation. This work explores TRIB1 regulation, focusing on its post-transcriptional aspects. In 3 distinct model systems (HEK293T, HeLa and arterial smooth muscle cells) TRIB1 was undetectable as assessed by western blot. Using recombinant TRIB1 as a proxy, we demonstrate TRIB1 to be highly unstable at the protein and RNA levels. By contrast, recombinant TRIB1 was stable in cellular extracts. Blocking proteasome function led to increased protein steady state levels but failed to rescue protein instability, demonstrating that the 2 processes are uncoupled. Unlike as shown for TRIB2, CUL1 and TRCPβ did not play a role in mediating TRIB1 instability although TRCPβ suppression increased TRIB1 expression. Lastly, we demonstrate that protein instability is independent of TRIB1 subcellular localization. Following the identification of TRIB1 nuclear localization signal, a cytosolic form was engineered. Despite being confined to the cytosol, TRIB1 remained unstable, suggesting that instability occurs at a stage that precedes its nuclear translocation and downstream nuclear function. These results uncover possible avenues of intervention to regulate TRIB1 function by identifying two distinct regulatory axes that control TRIB1 at the post-transcriptional level.

## Introduction

The first *tribbles* protein was identified in *Drosophila* as a protein required for oogenesis [1]. By comparison, higher eukaryotes express three distinct *tribbles*, TRIB1, 2 and 3 which are characterized by a relatively well conserved kinase-like domain (40–70% identity) and variable N and C-terminal regions [2]. Whether the *tribbles* are completely inactive as kinases is still unresolved as recent evidence suggests that TRIB2 possesses low nucleotide binding properties and a weak kinase activity at least *in vitro*, although structural data suggest that this ability may be lost in TRIB1 [3,4].

*Tribbles* have been implicated in multiple types of cancer. Early studies demonstrated that the *tribbles* are associated with myeloid leukemia [5,6] and a gain of function TRIB1 promotes leukemic transformation [7]. TRIB1 is also strongly upregulated in pancreatic cancer where it supports proliferation and survival [8]. In addition to its contribution to cancers, genome-wide-association studies (GWAS) have uncovered a functional association of the *TRIB1* locus with lipid traits, cardiovascular disease (CAD) as well hepatic steatosis, the accumulation of fat in the liver [9–12]. Experiments in animal models support the statistical association of TRIB1 with disease where altering *Trib1* expression affects lipid and glucose homeostasis [11,13,14].

A prominent hypothesis to account for the role of TRIB1 in disease involves the degradation of C/EBP proteins by TRIB1 [5,14–16]; this process is conserved in Drosophila where degradation of the C/EBP ortholog (*slbo*) is stimulated by *tribbles* [1]. TRIB1 could serve as a scaffold for the assembly of C/EBPA and/or C/EBPB and COP1, an E3 ligase, thereby promoting the degradation of these C/EBPs via the Ubiquitin Proteasome System (UPS) [17]. Whereas the UPS has been shown to play a prominent role in mediating TRIB1 action in the degradation of the CEBPs, its role in the regulation of TRIB1 itself remains to be explored.

While a clearer picture of TRIB1 function is slowly coming into focus, regulation of the *tribbles* and TRIB1 in particular, remains largely unexplored. The available data hint that it is likely complex. For one, *TRIB1* is expressed from an unstable RNA [18,19]. In addition the *TRIB1* gene contains a long 1.5 Kbp 3' untranslated region (UTR), quite conserved in parts, that undergoes miRNA regulation [20]. The importance of mammalian *tribbles* to both physiological and pathological processes emphasizes the need to clarify and elucidate the mechanisms regulating the *tribbles* and *TRIB1* in particular.

Prior work from our laboratory demonstrated the importance of transcriptional regulation in the response of the *TRIB1* gene to mitochondrial stressors [18]. However, TRIB1 could not be detected at the protein level, despite its wild-type sequence and significant RNA levels, suggesting that it might be suppressed post-transcriptionally [21]. Genome-wide analyses indicate that concentrations of mRNA and protein correlate by about 40%, indicating that post-transcriptional regulation plays a crucial role in controlling net protein output [22]. Indeed, mere presence in the cytosol does not guarantee translation as certain conditions elicit the redistribution of RNAs to regions for temporary storage [23]. Translation requires initiation, elongation and termination steps, all of which provide potential inroads for intervention should the situation demand it [24,25]. For example, p53 output can be enhanced by shifting the p53 transcript from ribosome-poor to ribosome-rich polysomes [26]. The net result is that translation efficiency, defined by the number of protein units per mRNA molecule, can vary substantially [27,28].

This work aims to provide a more comprehensive view of TRIB1 regulation, focusing on identifying processes regulating the stability of TRIB1 at the RNA transcript and protein levels, with the expectation that this knowledge should help identify potential avenues for disease prevention and intervention.

## Materials and Methods

### Western blotting

Lysates were obtained by incubating cells for 5 min in lysis buffer (20 mM HEPES, 0.13 M NaCl, 5 mM EDTA, 1% TRITON X-100) containing phosphatase and protein inhibitor cocktails (PhoSTOP and EDTA-free cOmplete protease inhibitors, Roche), unless specified. Typically, SDS-PAGE was performed using 8% acrylamide gels on 30 μg of lysates (assayed using Bradford reagent) per sample. Proteins were transferred to nitrocellulose for 1 h and efficiency was estimated using Ponceau and destained in PBS. Membranes were then blocked for 1 h in 5% skim milk or Odyssey blocker prior to detection, with similar results. All primaries were used at a concentration of 0.5 μg/ml while fluorescent secondary antibodies (LICOR) were used at 1:20K dilutions. Imaging was performed on an Odyssey system (LICOR). Even loading was confirmed using Tubulin β (TUBB) detection. Target quantifications are normalized to TUBB and are representative of 3 distinct biological replicates. Primary antibodies used are listed in the S1 Supplementary Materials section.

### Cell culture and treatments

HeLa and HEK293T were obtained from ATCC. Stable cell lines overexpressing TRIB1, derived from HeLa and HEK293T, were selected in high glucose DMEM (Gibco) supplemented with 10% FBS, Penicillin-Streptomycin and either 2 μg or 4 μg/ml of puromycin respectively. Smooth muscle cells (lot HITC6), derived from primary cultures of thoracic arterial cells, were obtained from Dr. JG Pickering [29] and were maintained in SmGM-2 medium (Lonza) supplemented with 5% FBS, insulin, hFGF-B, GA-1000, and hEGF. Infected SMCs were selected with 2 μg/ ml of puromycin. For the maintenance of resistant cells, puromycin concentration was halved. Cycloheximide (10 μg/ml), ACTD (5 μg/ml), MG132 (20 μg/ml) and Bortezomib (10 μg/ml) were added for 5 h unless mentioned otherwise. Vehicle (DMSO or H_2_0) was added at 0.1% or less. Lentiviral particles were purified from the supernatant of HEK293FT cells (Thermo Fisher Scientific) transfected with pLVX, pSPAX2 and pMD2.G (Addgene). For transient transfections, 1 μg DNA was complexed with 3 μl of Fugene 6 in optimem and added to cells maintained in 12 well plate wells for 48 h. For microscopy, cells were seeded on glass coverslips 24 h before transfection. For post-lysis stability assays, lysates of stable cell lines obtained with unsupplemented lysis buffer were combined with an equal volume of assay buffer (50 mM Tris-HCl, 10 mM MgCl_2_, 1 mM DTT, pH 7.9) and incubated for up to 18 h at 37°C.

### Molecular Biology and cloning

#### Quantitative Real-Time RT-PCR

RNA was isolated using the Roche High Pure RNA isolation kit and reverse transcribed using the Transcriptor First Strand cDNA synthesis kit (Roche Diagnostics) using a combination of oligo-dT and random hexamers. The resulting cDNA was analyzed by quantitative PCR on a LightCycler 480 using the Roche LightCycler 480 SybrGreen I Master mix (Roche). Cp values are normalized to PPIA levels. Oligonucleotides used are listed in the S1 Supplementary Materials section.

#### Cloning and deletions

Cloning was performed using standard molecular biology tools; olignucleotides are listed in the S1 Supplementary Materials section. TRIB1 was amplified from a construct obtained from Origene (pCMV6-XL5TRIB1). The open reading frame was amplified by PCR using Vent polymerase (New England BioLabs) and transferred in either pLVX for lentiviral expression or in either pCMV-Tag2 (Agilent) or pECFP-C1 (Clontech) for transient transfection of proteins containing N-terminal FLAG tag or eCFP tags, respectively. Deletions were performed using the Q5 polymerase (New England BioLabs). All constructs were verified by Sanger sequencing.

### Microscopy

Cells were seeded on glass coverslips and fixed for 15 min in 4% formaldehyde in Phosphate Buffered Saline (PBS). Cell permeabilization was achieved with PBS containing 0.1% TRITON X-100 for 15 min. Detection was performed by incubating with primary antibodies at 1:500 dilutions and secondaries (Jackson laboratories) at 1:2000 for 1 h in PBS. Four washes of 5 minutes in PBS were performed after each incubation. Cells were mounted in DakoCytomation Fluorescent Mounting Medium supplemented with Hoechst 33342. Microscopy was performed using a 100 X Oil Immersion lens on an Olympus confocal microscope. Images were obtained with the FluoView 3.1 software.

## Results

### *TRIB1* mRNA expression in model systems

**Fig 1:**
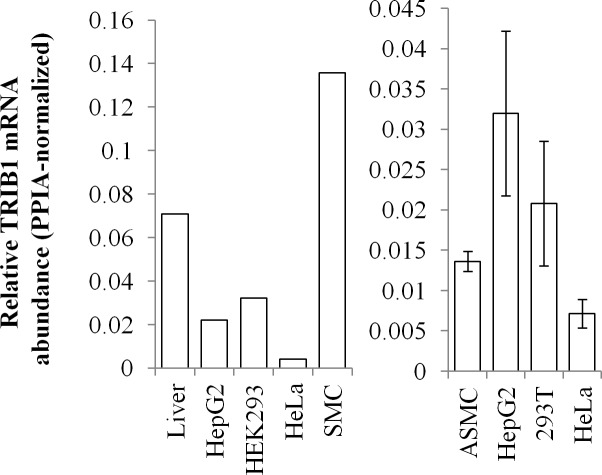
Endogenous *TRIB1* is expressed at low levels in several cell models. Left, RNA quantification by RNAseq calculated from to the Human Protein Atlas portal data, using PFKM counts for the indicated tissue (SMC, Liver) or cell lines (HepG2, HEK293). Right, RNA quantification by qRT-PCR in the indicated cell models. Abundance is normalized to PPIA. Quantification of 3–6 experiments is shown (± 95% CI).

Public databases of TRIB1 expression were consulted to identify potential cell systems amenable to study. According to the Protein Atlas (http://www.proteinatlas.org/), TRIB1 is widely expressed, including the commonly used cell lines HeLa and HEK293 (Fig 1, left). Another cell line of interest, in view of the role of demonstrated importance of *TRIB1* in liver function and its widespread use as a liver model, is the HepG2 hepatocarcinoma. While liver has a relatively high expression with levels of approximately 7% those of PPIA, HepG2 cells has slightly lower levels, approaching 2%. Our own qRT-PCR quantifications matched closely the database estimates for cell lines (Fig 1, right) with levels relative to PPIA in the 0.5–4% range. By contrast when we compared these levels to arterial smooth muscle cells (ASMC), quite distinct results were obtained with levels of 1.3% departing significantly from the Protein Atlas value of 14% for smooth muscle. These findings indicate widespread but variable expression of *TRIB1*.

### *TRIB1* mRNA is unstable in HeLa and ASMC

**Fig 2:**
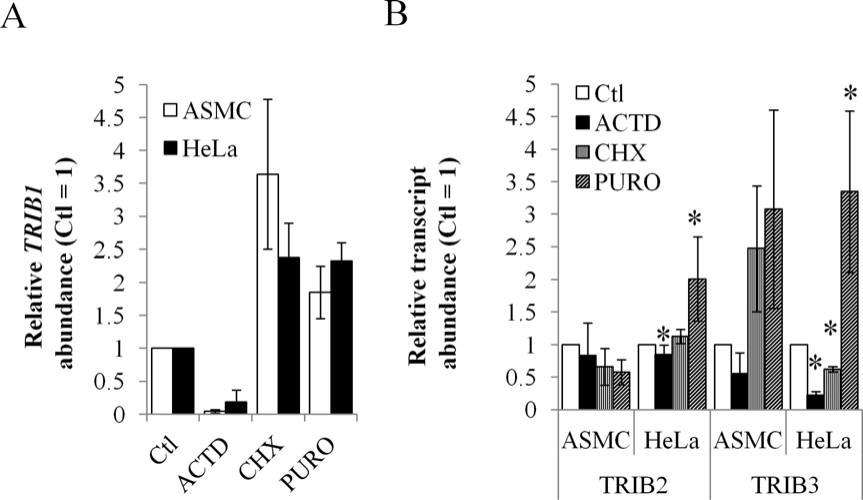
*TRIB1* transcript is unstable and increased by translation inhibitors. A, RNA from HeLa cells and ASMC treated with the indicated drugs for 5 h were harvested for quantification by qRT-PCR using primers specific for *TRIB1*. Values are first normalized to the housekeeping gene *PPIA* and then expressed relative to the Ctl values (Ctl = 1). B, as in A except that *TRIB2* and *TRIB3* transcripts were measured by qRT-PCR. * indicates statistical difference from control (p < 0.05, 2-tailed unpaired Student’s t- test) while in A, all changes were significant relative to the control. Error bars represent 95% confidence intervals.

Since we hypothesized that transcript instability might play an important role in TRIB1 expression, we measured transcript stability of *TRIB1* in 2 of these model systems, ASMC and HeLa cells. These cells were selected as primary and transformed models, respectively. HeLa cells have been used to investigate TRIB1 previously and were preferred for consistency [6,30,31]. As observed in HepG2 previously, *TRIB1* transcripts in both cell types were unstable: Actinomycin D (ACTD) treatment led to >80% suppression of *TRIB1* within 5 h (Fig 2A).

The contribution of translation to RNA stability was examined next, using cycloheximide (CHX) and puromoycin (PURO). Puromycin causes premature translation termination and detaches ribosomes from the mRNA while cycloheximide freezes ribosome/RNA complexes during elongation. Interestingly both treatments, despite opposite modes of action, increased *TRIB1* mRNA abundance 2-5-fold (Fig 2A), suggesting that the increase is the result of increased transcription rather than RNA stabilization. To determine the relative roles of transcription vs translation in this process, HeLa cells were co-incubated with ACTD and/or CHX, with the expectation that putative RNA stabilization should be insensitive to ACTD. As observed in HeLaT1, CHX alone increased *TRIB1* in HeLa cells as well. Inclusion of ACTD completely abrogated the CHX response, suggesting that CHX enhances *TRIB1* transcription in HeLa cells (S1 Fig).

As the 3 human *tribbles* evolved from a common ancestor and may share common regulatory mechanisms, we also examined the other 2 *tribbles* (Fig 2B). Controls, performed in HepG2 hepatoma cells, confirmed the specificity of the PCR primers used (S2 Fig). In HeLa and ASMC cells, *TRIB3* and *TRIB1* mRNA were present at comparable levels, while *TRIB2* was significantly less expressed (typical crossing points (Cp) values of 23–25 for *TRIB1* and *TRIB3* vs 28–30 for *TRIB2*). As assessed by their sensitivity to ACTD, *TRIB3* appeared somewhat unstable, although less so than *TRIB1*, while *TRIB2* was more stable. Translation inhibitors appeared to affect all 3 transcripts although only a subset of the changes reached statistical significance over 3 experiments.

### Endogenous TRIB1 is undetectable by western blot in HeLa and 293T

**Fig 3:**
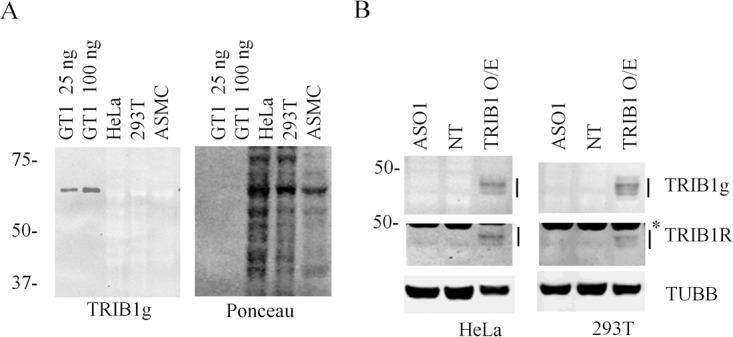
Endogenous TRIB1 is undetectable by straight western blot. A, HeLa, 293T and ASMC lysates were lysed in IP buffer and resolved by SDS-PAGE. Purified GST-TRIB1 fusion (GT1) protein was included as positive control. B, HeLa and 293T cells treated for 48 h with a TRIB1 (ASO1) or a control (NT) antisense oligonucletide were lysed in IP buffer supplemented with protease and phosphatase inhibitor cocktails, resolved by SDS-PAGE and analyzed by western blotting using 2 distinct TRIB1 antibodies. Cell lines stably transduced with TRIB1 and lysed under the same conditions were used as positive control. TRIB1 bands (indicated by side bars) had apparent masses ranging from 43–48 kDa. * indicates residual TUBB signal.

Prior work from our laboratory showed that TRIB1 was undetectable by western blot in HepG2 cells, as no siRNA sensitive band could be identified [18]. In addition purification of a ~ 45 kDa TRIB1 antibody-reacting band led to the identification of a spurious target. Modifications to the protocol were implemented to eliminate non-specific background, most notably the exclusion of detergents from the detection procedure. Using this modified protocol, background signals from the 3 model systems above revealed little to no signal, visible as negative staining bands which reflected general protein abundance (see Ponceau stain), whereas a strong signal was obtained from recombinant (GST)TRIB1 (Fig 3A). Titration experiments indicated the detection limit to be in the order of a few nanograms (S3 Fig), with the goat host antibody showing the greatest sensitivity.

### Establishment of model systems stably expressing TRIB1

To ascertain that the extraction conditions were sufficient to extract TRIB1 from the cellular milieu, stable populations of TRIB1 expressing HeLa and 293T cells were generated using recombinant lentiviral particles harboring the open reading frame of TRIB1. Infection resulted in 67-fold (± 13) and 153-fold (± 46) upregulation in *TRIB1* message for 293T and HeLa cells respectively. Upon examination of the HeLa stable pool by immunocytochemistry, TRIB1 expression was seen as diffuse, with some granularity (S4 Fig). Expression was exclusively nuclear with variable levels across the cell population, as expected from a non-clonal population. Western blot analyses of lysates derived from these transduced HeLa or 293T (HeLaT1 and 293T1) cells, but not control lysates, showed multiple, closely spaced bands with masses ranging from 43–48 kDa (Fig 3B).

### TRIB1 protein is unstable *in vivo* but stable *in vitro*

**Fig 4:**
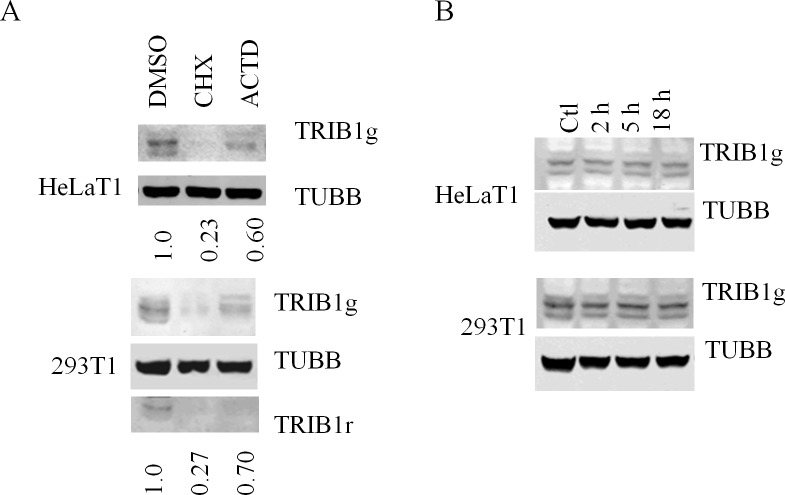
TRIB1 is unstable in HeLaT1 and 293T1 *in vivo* but stable *in vitro*. A, stably integrated pools of cells were treated with CHX or ACTD for 5 h and analyzed by western blot using TRIB1(TRIB1g (goat) or TRIB1r (rabbit)) and TUBB specific antibodies. Data are representative of 3 experiments. Quantification presented is for the TRIB1g and TUBB only. B, TRIB1 stable cells were lysed in IP buffer with no additives, diluted in assay buffer and incubated at 37°C for the indicated time. Western blot was performed with the indicated antibodies.

Stability of the recombinant protein was then assessed in the 293T1 and HeLaT1 systems. As shown in Fig 4A, the multiple bands were sensitive to translation (CHX) and transcription (ACTD) inhibitors. Addition of CHX led to >70% reduction in TRIB1 signal in both systems. By comparison, ACTD seemed less potent with a more modest 30–40% reduction after a 5 hour period. TRIB1 reacting bands were also visible with a distinct primary antibody indicating that epitope masking is unlikely to account for the signal reduction. Time course experiments performed in HeLaT1 indicated that the signal had a half-life of ~ 90 min in response to CHX (S5 Fig).

These experiments indicate that the protein is unstable *in vivo*. To obtain an estimate of the stability of mature TRIB1, lysates obtained from 293T1 and HeLaT1 were diluted in a reaction buffer and incubated *in vitro* at 37°C; protease and phosphatase inhibitors were deliberately excluded to help identify putative proteolytic activity. For up to 18 h incubation, TRIB1 was found to be unaltered in both cell types (Fig 4B). Thus, under these assay conditions at least, TRIB1 appears remarkably stable.

### Impact of transcription and translation inhibitors on recombinant TRIB1

**Fig 5:**
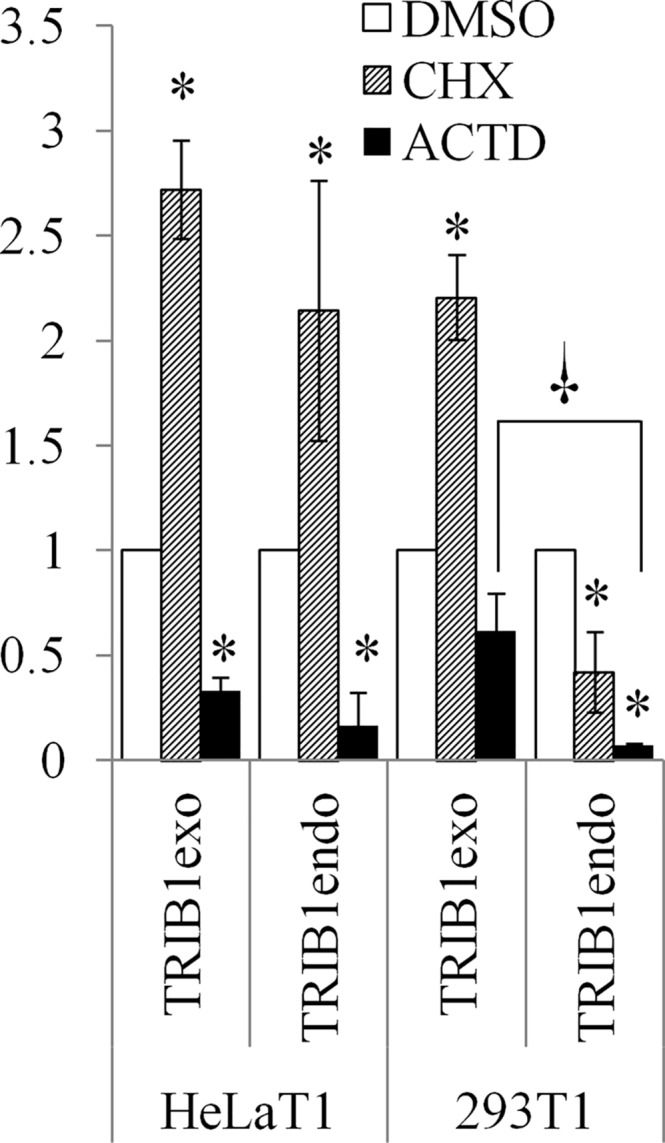
Exogenous transcript is upregulated by CHX and is more stable than the endogenous. Stable pools of HeLaT1 and 293T1 were analyzed by qRT-PCR using primer pairs specific for either the exogenous (transduced) *TRIB1* or the endogenous transcript. * indicates statistical significance versus DMSO while † indicates statistical significant difference between endo and exo signals (p < 0.05, 2-tailed unpaired Student’s t- test). Values are normalized to the DMSO control.

The relative inefficiency of TRIB1 protein suppression by ACTD contrasts with the instability of the endogenous transcript to this drug (Fig 2A) which, in conjunction with the short half–life of TRIB1, would be predicted to lead to the rapid disappearance of the signal. This suggested that the exogenous transcript was more stable, i.e. less ACTD sensitive, than the endogenous. As the PCR primer pair used earlier for qRT-PCR did not distinguish between the endogenous and exogenous forms, primer pairs that could discriminate between the two forms were designed, taking advantage of distinct 3’ UTRs. Analysis of RNA derived from ACTD-treated 293T1 and HeLaT1 confirmed that *TRIB1* was more resistant than the endogenous form to ACTD (Fig 5), to an extent consistent with the impact of the drugs visible at the protein level (Fig 4). This was particularly evident in 293T cells, although the same trend was observed in HeLa cells.

Contrasting with its effect at the protein level, blocking translation increased recombinant *TRIB1* message in both cell systems (Fig 5). As for the endogenous message, results are more nuanced, with an increase in endogenous message, consistent with its impact on wild-type HeLas (Fig 2A), but a decrease in the 293T1 endogenous message. Thus both transcript species are affected similarly in response to CHX in HeLaT1 but in opposite direction in 293T1 cells.

### Proteasome regulates steady state TRIB1 level but not its instability

We reasoned that the instability of the TRIB1 protein might stem from proteasome-mediated degradation. Indeed, TRIB2’s stability has been shown to be the under the control of proteasome-mediated degradation [32]. Furthermore, rapid degradation of newly synthesized polypeptides via the ubiquitin-proteasome machinery (UPS) is common [33].

**Fig 6:**
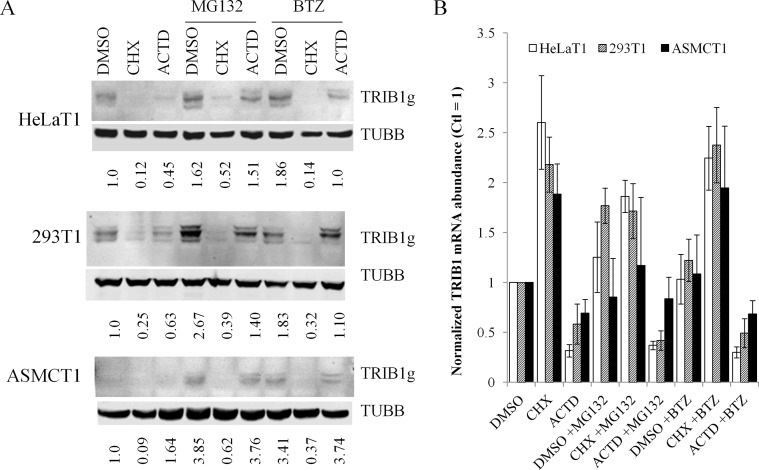
Proteasome inhibitors increase TRIB1 but do not prevent TRIB1 destabilization in stably transduced cells. Cells were treated for 5 h with vehicle, CHX or ACTD, in combination with MG132 (20 μM) or Bortezomib (BTZ, 10 μM), as indicated. A, Lysates were subjected to western blot using the indicated antibodies. B, mRNA was isolated and quantified by qRT-PCR for *TRIB1*. Values are normalized to vehicle alone (DMSO) and represent the mean of biological triplicates ± S.D.

To test the contribution of the UPS system to TRIB1 degradation, 2 structurally distinct proteasome inhibitors, bortezomib (BTZ) and MG132 were included with the CHX treatment (Fig 6A). Inclusion of the proteasome inhibitors increased TRIB1 protein level in both HeLaT1 and 293T1. A similar pattern was observed in ASMC that were generated by transduction of the *TRIB1* coding sequence to examine regulation in a non-immortalized cell model. TRIB1 protein expression was particularly low in ASMC, despite *TRIB1* transcript levels being intermediate to those of 293T1 and HeLaT1; TRIB1 was only clearly visible after treatment with proteasome inhibitors. Parallel experiments in uninfected controls showed no detetectable protein, even after treatment with MG132 (data not shown). Importantly however, in all 3 systems the inhibitors failed to rescue the CHX-mediated downregulation of the TRIB1 protein. Thus, inhibiting the proteasome increases total TRIB1 levels but has no effect on its instability.

These changes could be the result of increased *TRIB1* transcript. MG132, but not BTZ, did increase *TRIB1* transcript levels in 293T cells significantly, with a similar trend in HeLa cells (Fig 6B). By contrast, no such increase was observed in ASMCs, despite increases in TRIB1 protein (Fig 6A). Thus while proteasome inhibition can increase *TRIB1* mRNA abundance under some conditions, transcript upregulation is not required for the augmented protein level.

### The *TRIB1* UTRs play no evident role in the stabilization of TRIB1 in HeLa cells

**Fig 7:**
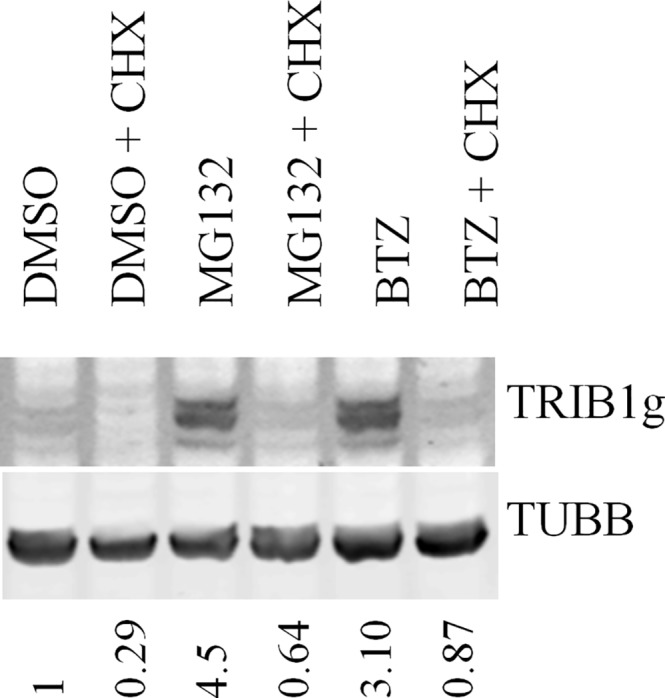
Minimal impact of untranslated regions on TRIB1 stability. Stable HeLa cell lines expressing TRIB1 with native 5’ and 3’ UTRs (HeLaT1UTR). Cells were treated with MG132 (20 μM) or BTZ (10 μM) or vehicle (0.1% DMSO) in the presence or absence of CHX for 5 h. Cells were lyzed and analyzed by western blotting using TRIB1 or TUBB antibodies. Data are representative of 3 biological replicates.

*TRIB1* contains long untranslated regions (UTRs) with likely functional correlates. Indeed its 3’UTR is regulated by miR-224 in prostate cancer models [20]. To evaluate the contributions of the *TRIB1* UTRs to its expression, *TRIB1* was amplified together with its UTRs from a commercially available *TRIB1* construct containing a partial 5’and a complete 3’ UTRs. Unfortunately attempts to isolate the longest reported form (corresponding to NM_025195.3) by RT-PCR from several primary tissues failed. Nevertheless, inclusion of the UTRs nearly tripled the size of the *TRIB1* expression cassette from 1.1 kb to 3.1 kb. First, the extended *TRIB1* construct was introduced in HeLa cells and *TRIB1* RNA abundance following a 5 h ACTD regimen was assessed. Note that the transduced transcript and endogenous *TRIB1* transcripts could not be distinguished using our qRT-PCR assay. Total TRIB1 was increased ~40-fold following TRIB1 transduction, so that the transduced transcript contributes to most of the measured signal. ACTD treatment led to the rapid disappearance of *TRIB1* with residual levels (after 5 h) comparable (33 ± 1% of DMSO signal (n = 2)) with the transduced ORF only transcript. Thus the UTRs do not appear to contribute significantly to the stability of the *TRIB1* transcript. At the protein level, the TRIB1 signal in transduced cells was faint (Fig 7), possibly reflecting lower transcript abundance (~4-fold lower than HeLaT1) and was sensitive to CHX. As seen with the ORF only construct, MG132 and BTZ increased TRIB1 steady state levels but failed to stabilize the protein (Fig 7). Thus the UTRs have no noticeable impact on the regulation of TRIB1 instability.

### Indirect implication of βTRCP but not CUL1, in the regulation of TRIB1 abundance

**Fig 8:**
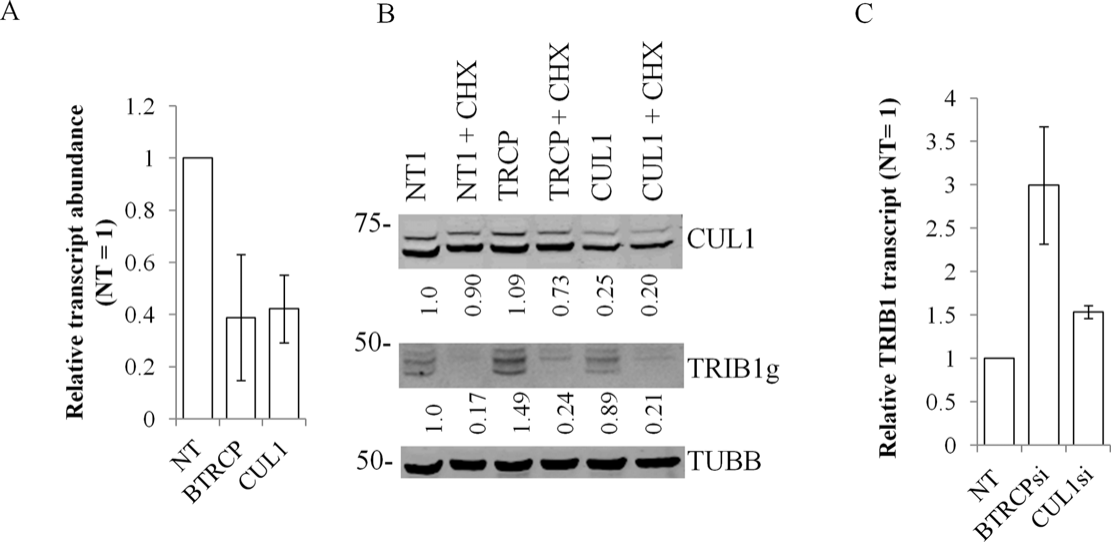
Suppression of βTRCP increases *TRIB1* expression but does not affect its instability. HeLaT1 cells incubated for 48 h with siRNA targeting CUL1, βTRCP or a non-target control were treated with vehicle (H_2_0) or CHX for 5 h. Parallel lysates were then analyzed by western blot and qPCR. A, qRT-PCR of RNA isolated from cells treated with the indicated siRNAs for 48 h. Quantification of corresponding transcript is shown, normalized to the non-target oligonucleotide (NT). B, western blot of extracts from silenced cells. Data shown are representative of 3 biological replicates. C, βTRCP and CUL1 suppression increase *TRIB1* transcript abundance. qRT-PCR of *TRIB1* in cells suppressed for the indicated siRNAs. In A and C, data represent the average of 2 biological replicates ± S.D.

Recently TRIB2 expression has been shown to be negatively regulated by the SCF^βTRCP^ complex: suppression of either Cullin1 (CUL1) or βTRCP led to the stabilization and increased expression of TRIB2 protein [32]. Cells contain several types of Skip-Cullin-F-box complexes that target cognate targets for protein degradation via the UPS [34]. Interestingly the region responsible for the βTRCP F-box protein interaction on TRIB2 shows a good conservation with TRIB1 indicating that TRIB1 may be similarly regulated. While our data are inconsistent with a prominent role of the UPS in mediating TRIB1 instability, the SCF^βTRCP^ could nonetheless regulate its steady state level. The contributions of CUL1 and βTRCP were tested by suppressing their expressions in HeLaT1 cells by siRNA treatment. Treatment led to a ~ 50% reduction of *CUL1* or *βTRCP* transcripts (Fig 8A). Parallel western blots showed a comparable reduction in CUL1 at the protein level. Unfortunately, attempts to detect βTRCP were unsuccessful. These reductions were not accompanied by reduced sensitivity to CHX, indicating that neither protein is likely to regulate *TRIB1* transcript stability (Fig 8B). Nonetheless, βTRCP suppression increased TRIB1 protein abundance by ~50%, consistent with a role in repressing TRIB1 function. Parallel examination of the *TRIB1* transcript uncovered a 2-4-fold increase following βTRCP knockdown which could account for this increase (Fig 8C).

### TRIB1 NLS is located near its N-terminus

Several mechanisms could account for the *in vivo* destabilization. The protein could be unstable and degraded while performing its function or could be degraded at an earlier stage, for instance co-translationally or prior to its import into the nucleus. We reasoned that this model could be tested by changing the cellular localization of TRIB1 and measuring the stability of the resulting polypeptide in the presence of CHX.

**Fig 9:**
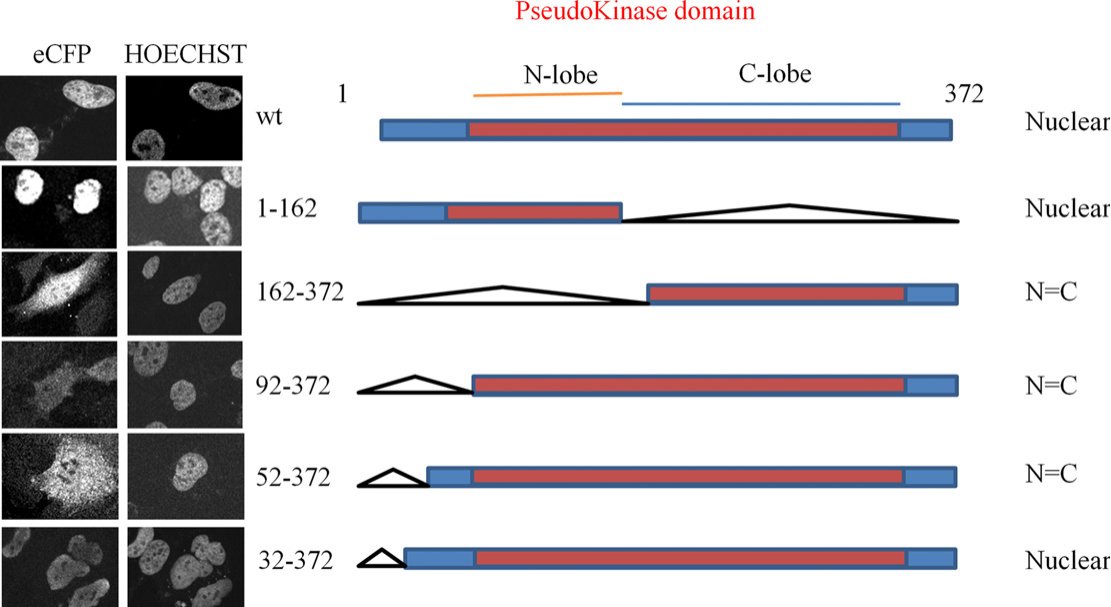
TRIB1 NLS is located near its N terminus. Variants of TRIB1 fused C-terminal to eCFP were expressed in HeLa cells for 24 h, fixed, permeabilized and subjected to fluorescence confocal microscopy using a 440 nm laser. Hoechst 33342 was included to visualize the nucleus. Data represent 2 independent experiments. Schema of the deletions is shown on the right.

With the aim of generating mutants of TRIB1 that show no preferential localization or preferentially relocate to the cytosol, mapping of the localization site(s) of TRIB1 was undertaken. Analysis of TRIB1 sequence with cNLS mapper (http://nls-mapper.iab.keio.ac.jp/cgi-bin/NLS_Mapper_form.cgi), which identifies putative classical mono- and bipartite NSLs, a strong monopartite NLS was predicted to be in the pseudo-kinase domain, centered on residue 170 (score of 9.5) and a weaker bipartite (score of 7.5) spanning AA 27–51. Putative NLSs containing regions were mapped by performing deletions in the context of eCFP-TRIB1 fusions that were transiently transfected in HeLa cells (Fig 9). A construct spanning AA 162–372 only resulted in the pancellular distribution as expected from passive movement of the GFP-TRIB1 truncation through the nuclear pore complex, demonstrating that the N terminus is necessary for nuclear targeting. Indeed, expression of AA 1–162 resulted in nuclear localization, thus identifying the N-terminal region as sufficient for nuclear localization. Further mapping refinements revealed that residues located in a region comprising AA 33–51 are needed for nuclear localization (Fig 9).

**Fig 10:**
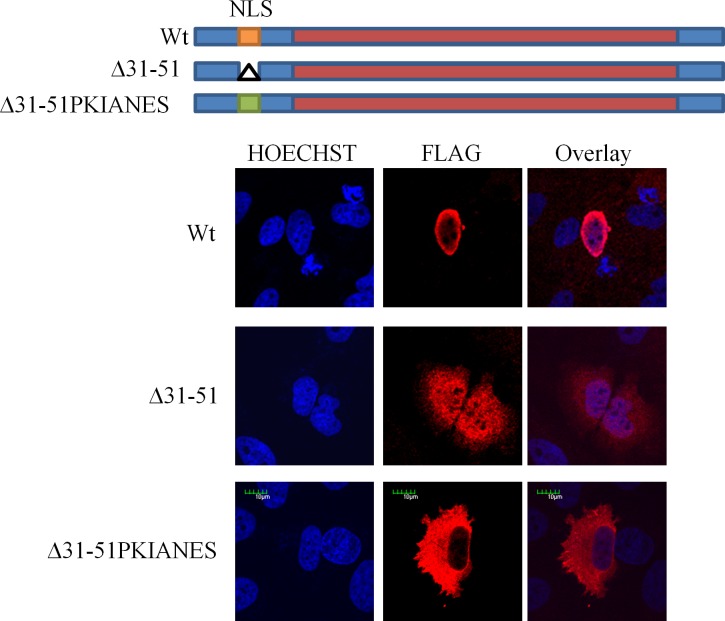
AA31-51 are required for nuclear localization of TRIB1. Top, schema of the deletions and insertions introduced in FLAG-tagged TRIB1. Bottom, immunofluorescence analysis of HeLa cells transfected for 48 h with the indicated TRIB1 mutants. FLAG tagged TRIB1 expressing cells were fixed, permeabilized and examined by confocal microscopy using an antibody specific for the FLAG epitope and an anti-mouse secondary (Alexa 647 goat anti-mouse, Molecular Probes). Nuclei were counterstained with Hoechst 33342.

Removal of AA 33–51 in the context of a FLAG tagged TRIB1 constructs led to the pancellular redistribution of TRIB1, confirming that they are required for the preferential redistribution of TRIB1 to the nucleus (Fig 10). As residual nuclear presence, presumably from passive NLS-independent diffusion, might have complicated possible downstream interpretation, an additional construct was generated by substituting the Protein Kinase (CAMP-Dependent, Catalytic) Inhibitor Alpha (PKIA) nuclear export signal (NES) in place of the NLS [35]. Substituting the NLS with the strong PKIA NES mistargeted TRIB1 to the cytosol (Fig 10).

### TRIB1 instability is independent of its cellular localization

**Fig 11:**
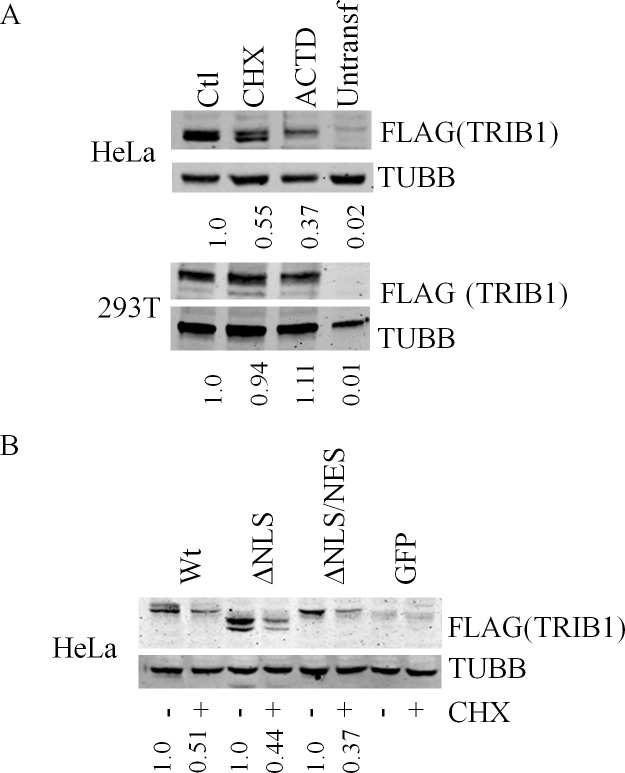
TRIB1 instability is independent of its subcellular localization. A, cells were transfected for 48 h with constructs driving FLAG-TRIB1 expression. Cells were then treated with the indicated drugs for 5 h, harvested and the lysates analyzed by western blotting. B, HeLa cells were transiently transfected with constructs coding for NLS-targeted FLAG tagged TRIB1 (48 h), Wt FLAG tagged TRIB1 or a GFP control plasmid and treated with CHX (10 μg/ml) for 5 h. Lysates were resolved on a 4–15% gradient gel (A) or a 8% SDS-PAGE (B), transferred to nitrocellulose and probed with FLAG or βTubulin (TUBB) antibodies as indicated.

Hela cells were transiently transfected with the engineered FLAG TRIB1 constructs whose stabilities were assessed in the presence of CHX. Pilot experiments indicated that 293T cells were unsuitable for these experiments as transiently transfected TRIB1 was found to be CHX-(and ACTD-) insensitive in that system, for reasons that are unclear but may have to do with high expression level (Fig 11A). In comparison, when transiently introduced in HeLa cells, TRIB1 was more sensitive to CHX, albeit less so than transduced TRIB1, with TRIB1 showing only ~ 50% reduction (vs > 80% in HeLaT1) following a 5 h regimen with CHX. Upon their introduction in HeLa cells, CHX affected all 3 variants similarly (Fig 11B). Thus, TRIB1 is unstable as a result of events occurring during or early after protein synthesis, before the protein enters the nucleus.

## Discussion

This report demonstrates that TRIB1 is an unstable protein expressed from an unstable transcript whose steady state can be increased by the proteasome, a major eukaryotic system for targeted intracellular protein degradation [17,36]. These changes do not require increased RNA abundance, although increases were observed under certain conditions, pointing to post-transcriptional regulation. Proteasome involvement is consistent with the role of TRIB1 as a COP1 adaptor protein. TRIB1 could be ubiquitylated by COP1/CEBP related processes and degraded by the proteasome.

**Fig 12:**
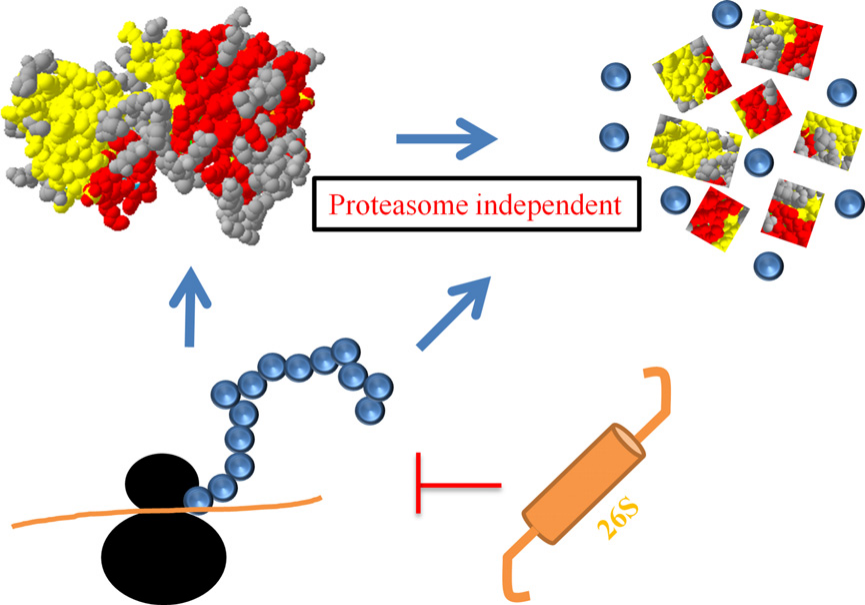
Model of TRIB1 regulation. TRIB1 is rapidly degraded in the cytosol during or after synthesis by proteasome-independent processes that remain to be identified. As for the proteasome, it curbs TRIB1 expression by reducing net translational output. The protein Ball and Stick structure shown corresponds to TRIB1 (pdb5cem) and was generated with the Swiss-PdbViewer; β sheets residues are in yellow and α helical residues in red while unstrunctured residues are displayed in gray.

The proteasome however does not seem to play a role in the regulation of TRIB1 stability *per se*. Rather, our results indicate that TRIB1 instability can be controlled at an early point before TRIB1 enters the nucleus. This suggests co-translational regulation, but does not exclude a contribution of other downstream cytosolic events. We propose that TRIB1 can be regulated by 2 distinct translational events that can be distinguished based on their requirement for proteasomal activity (Fig 12). One, under kinetic regulation, controls the turnover rate of the protein. Whether this early regulatory node is linked to the short transcript half-life remains a matter of speculation but is consistent with these findings. This process is largely if not entirely proteasome-independent and as such does not meet the requirement of the classical translation quality control pathways mediated by heat shock proteins [37]. While stabilizing the TRIB1 protein might conceivably lead to increased total output, our proteasome experiments demonstrate that it is not a requirement, as the proteasome inhibitors did not prevent loss of TRIB1 following CHX treatment. Instead, these experiments point to a control that increases the steady state level of TRIB1 without changing its basic instability. This is unlikely to be achieved solely by increased transcripts as our results exclude a consistent (i.e. MG132 and BTZ-dependent) increase in total RNA following short term treatment with these inhibitors. We did however observe that longer (16 h) MG132 and BTZ regimens led to increased transcript and protein abundance from the TRIB1UTR and TRIB1 constructs (data not shown), which probably reflect increased transcriptional output secondary to cellular stress caused by prolonged UPS interference.

One can envision that blocking the proteasome pathway leads to a more efficient translation of existing *TRIB1* mRNA and, conversely, that these pathways normally restrain the translation machinery handling TRIB1. The UPS system plays a nuanced but pivotal role in the control of translation. Proteasome inhibition induces a stress response that includes a global reduction of translation activity as well as targeted increases in key stress-induced proteins including CHOP and ATF3/4 [38]. Under normal conditions however, important translation-initiation factors including eIF4G and eIF3A undergo UPS mediated degradation, inhibiting translation in the process [36].

This work addressed the role of UTRs in TRIB1 regulation only partially by demonstrating that these regions do not play a prominent role in regulating TRIB1 protein instability. This is not to be misconstrued as evidence that they play no role in regulating TRIB1. TRIB1 may be regulated at other levels, as seen with the UPS system. Given the important role UTRs have on RNA regulation [39], the role of these regions will need to be examined in greater depth. A recent report identified miR-224 as capable of suppressing TRIB1 protein level ~2-fold, although the underlying mechanisms were not examined [20]. Publicly available algorithms (e.g. http://www.microrna.org/) predict several putative miRNA binding sites in the 3’UTR but these theoretical predictions need to be experimentally validated. Previous work by Kiss-Toth et al that demonstrates a net (RNA) stabilizing role of the 3’UTR when overexpressed in *trans*, is certainly consistent with the role of a net destabilizing effect by the 3’UTR on TRIB1 mRNA [40].

Within the *tribbles* family, RNA instability is not unique to *TRIB1*. We demonstrate that *TRIB3* (but not *TRIB2*) is also sensitive to ACTD. This contrasts with prior findings where all 3 transcripts were unstable in mouse embryonic stem cells with half-lives of 0.9, 1.8 and 2.8 h for *TRIB1*, 2 and 3 respectively [19]. Hence these numbers agree well with ours with TRIB1 being the most unstable but differ with regard to TRIB2 stability. Presumably these differences reflect different cellular and/or species-specific functions which underscore that *tribbles* RNA stability has the potential to be regulated. At the protein level, preliminary experiments indicate that the TRIB3 protein is also sensitive to CHX (we have yet to examine TRIB2). Interestingly, genes characterized by unstable transcripts and proteins tend be regulatory genes, whose expressions need to be highly responsive to environmental cues [22]. As for TRIB2, work by Wilkin *et al* showed overexpressed TRIB2 to be highly labile in dog thyroid cells and undetectable after 1 h CHX treatment [41]. A recent report by Qiao et al also confirms that TRIB2 is unstable and that this instability is, in contrast to TRIB1, proteasome-dependent. More specifically in hepatocellular carcinoma Bel-7402 cells, TRIB2 instability requiresβTRCP and total levels are under the regulation of CUL1 [32,42]. These results clearly contrast with our findings on TRIB1 and underscore a fundamental difference between the 2 *tribbles*. Alternatively, the βTRCP regulatory axis might represent a cancer adaptation. Judging from the presented western blot evidence, it seems that the amount of TRIB2 is considerable in Bel-7402 and perhaps the SCF^βTRCP^ targets TRIB2 only when it is amplified. *Tribbles* amplification has been reported in some pancreatic cancer cell lines [8] and increased *tribbles* expression may represent a common strategy adopted by some cancerous cells to meet their altered metabolic demands.

Unlike our original hypothesis, *TRIB1* transcript instability does not solely account for the lack of detection seen in our model systems. Indeed, overexpressed TRIB1 was detected despite being sensitive to CHX. In this respect TRIB1 behaves like the majority of genes, where, unlike RNA abundance, RNA stability is a poor predictor of protein abundance [22]. Rather, the lack of detection of the endogenous signal may reflect low RNA abundance. Detection of TRIB1 was evident in the stable cells we generated but only after 60-200-fold overexpression of TRIB1, suggesting that the basal levels are very low. Extrapolating from the *in vitro* titration of the antibody, and assuming a linear relationship between the *TRIB1* RNA and protein amounts, approximate upper limits to the amount of cellular TRIB1 can be calculated. Using a conservative estimate of 10 ng of signal per 30 ug of cellular HeLaT1 extracts (corresponding to ~ 1 X 10^5^ HeLa cells), and the 150-fold RNA overexpression value, one can estimate upper limits in the amount of endogenous protein in HeLa, with as many as 9,000 molecules per average HeLa cell. This would place TRIB1 close to the median number of 18,000 copies per protein found in the average HeLa cell [43]. This represents upper limits and true amounts are likely much lower as mass spectrometry failed to detect TRIB1, 2 or 3 in HeLa extracts [43].

These observations highlight the low expression levels of TRIB1, at least in our model systems, but should not be interpreted as evidence for a lack of function. The *TRIB1* transcript is ubiquitously expressed, typically at low levels, with preferential expression in liver and lymphoid tissue (http://www.proteinatlas.org/ENSG00000173334-TRIB1/tissue). In HepG2 cells for instance where levels of *TRIB1* transcript compare with the 293T, we recently demonstrated that *TRIB1* suppression was associated with marked transcriptome alterations in primary human hepatocytes [21]. Rather, low expression suggests significant signal amplification is a prerequisite to function. This would be in line with transcriptional regulation at a few select loci or enzymatic functions via regulation of kinases for instance. Although a role as a kinase is inconsistent with the recently solved structure of TRIB1, TRIB1 could still interact and regulate the activity of kinases, as shown for MAP2K4 and MAP2K1 [31,44].

Our results identify the N-terminal region as required for nuclear import of TRIB1 in HeLa cells. According to publicly available data a second form of TRIB1 transcript, encoding AA 167–372 has been reported (NM_001282985.1). This form, possibly resulting from alternate splicing and transcription initiation sites corresponds for the most part to our fusion eCFP that was entirely cytosolic, leaving open the possibility that it performs a cytosolic function. This alternate form requires further validation however as its abundance is orders of magnitude lower than the canonical TRIB1 form (see publicly accessible GTEx portal data for TRIB1at http://www.gtexportal.org/home) and it may represent transcriptional noise. What about the other *tribbles*? According to cNLS Mapper, predictions for TRIB3 indicate the presence of a strong bipartite NLS spanning AA 15–37, that is consistent with its nuclear residence and is reminiscent of TRIB1. By contrast, no NLS, mono- or bipartite is predicted for TRIB2, in line with reports indicating that TRIB2 does not show preferential localization to the nucleus, and might actually be excluded in some systems [40,41,45]. A similar analysis of *Drosophila tribbles* fails to identify classical NLSs; *tribbles*, like TRIB2, has been reported to be present in the nucleus as well as the cytoplasm [1]. Thus the appearance of a bipartite NLS seems to be a recent addition that may have taken advantage of the poorly conserved and loosely structured N-terminal region to tailor certain *tribbles* for exclusive nuclear functions. TRIB2 (or indeed *tribbles*) could still perform nuclear functions since TRIB2 may be compact enough to diffuse passively through the nuclear pore complex into the nucleoplasm, a process that could be facilitated by putative non-classical NLSs [46].

Taken together these findings lead to the conclusion that interventions aimed at regulating TRIB1 should focus on modulating transcript levels, translation efficiency, protein stability or biological activity rather than affecting RNA stability. This could be achieved by altering its interaction interfaces with known effectors (e.g. MEK1, COP1, CEBPs). As TRIB1 could be tumorigenic [8,47], our results also suggest that treatment with proteasome inhibitors, which are currently being used as treatment for certain forms of cancer (e.g. Velcade®) [48,49], may benefit from adjunctive therapy with TRIB1 inhibitors or suppressors with the aim of offsetting associated TRIB1 level increases; the recently available structure of TRIB1 will be of considerable help in this matter [3].

Cancer amplification notwithstanding, a moderate increase in TRIB1 may indeed be beneficial. In a previous study we correlated a *TRIB1* genotype (rs2001844), associated with protection against CAD and decreased circulating triglycerides, with increased *TRIB1* expression. More specifically, *TRIB1* mRNA levels in the homozygous risk group were 20% lower than in the homozygous reference group [50]. To this day it remains unclear how this polymorphism located 30 kb away from the TRIB1 gene proper is impinging on these traits. That region harbors sequences with enhancer capabilities and encodes for a nuclear non-coding transcript (*TRIBAL*) that have the potential to affect net protein output by modulating *TRIB1* transcription [11,50]. However as these are regulatory events are confined to the nucleus they are unlikely to influence TRIB1 protein stability which we demonstrate to be controlled in the cytosol. While the CAD/dyslipidemia-associated etiology is unlikely to implicate changes in TRIB1 protein stability, increasing the latter modestly by targeted pharmacological interventions may still offer a means to harness the protective functions of TRIB1.

## Supporting Information

S1 Fig TRIB1 upregulation in response to CHX is secondary to increased transcription. TRIB1 transcript was measured in HeLa cells in response to CHX and/or ACTD by qRT-PCR. Cells were pre-treated with ACTD for 10 min prior to CHX addition, followed by a co-incubation for 5 h. All changes are statistically different from control (CTL). Inclusion of ACTD prevented the CHX-induced TRIB1 upregulation (4.3 vs 4.6%, p = 0.43). Biological replicates are distinct from those in Fig 2 and represent the average of 3 experiments. Error bars represent S.D.
(PDF)

S2 Fig Tribbles qRT-PCR controls. HepG2 cells were transfected with the indicated *tribbles* specific siRNA for 48 h and the levels of the *tribbles* mRNA quantified by qRT-PCR. Results represent the mean of 4 experiments (± 95% C.I).* indicates statistical significance (p < 0.05, Student’s unpaired, 2-tailed, t-test).
(PDF)

S3 Fig Antibody detection limit on SDS-PAGE is in the low nanogram range. Purified GST TRIB1 fusion proteins were isolated from BL21 bacteria and quantified relative to bovine serum albumin. Glutathione eluted proteins were diluted serially, resolved by SDS-PAGE and transferred to nitrocellulose. Western blot was performed using 0.5 ug of either rabbit or goat TRIB1-specific antibody and cognate secondaries (LI-COR; Donkey anti rabbit 680 and Donkey anti goat 800).
(PDF)

S4 Fig Immunofluorescence of HeLa and 29T cells stably transfected with TRIB1. HeLa and 293T cells were stably transduced with lentiviral particles encoding for TRIB1. Cells were fixed, permeabilized and blocked in 10% horse serum/ PBS. Microscopy was performed using either a donkey antibody targeted towards the C-terminus of TRIB1 (T1G) or the rabbit specific for an epitope contained within AA104-372 (T1R). Overlay of the 2 TRIB1 signals is shown on the right. Scale bar length corresponds to 10 μm.
(PDF)

S5 Fig Time course of TRIB1 signal in response to ACTD. HeLaT1 cells were treated with ACTD (5 μg/ml) for the indicated time. Graphical representation representing the average of 2 experiments (± S.D.) fit to an exponential regression is shown at the bottom; half-life is estimated at 94 min
(PDF)

S1 Supplementary Materials Details of oligonucleotides, antibodies and siRNA used in this work. (DOC)
