# Supplementary figures and images for: TRIB1 Is Regulated Post-Transcriptionally by Proteasomal and Non-Proteasomal Pathways
Source: PLoS One. 2016 Mar 28;11(3):e0152346. doi: 10.1371/journal.pone.0152346 (PMC4809572; doi:10.1371/journal.pone.0152346)

S1 Fig.

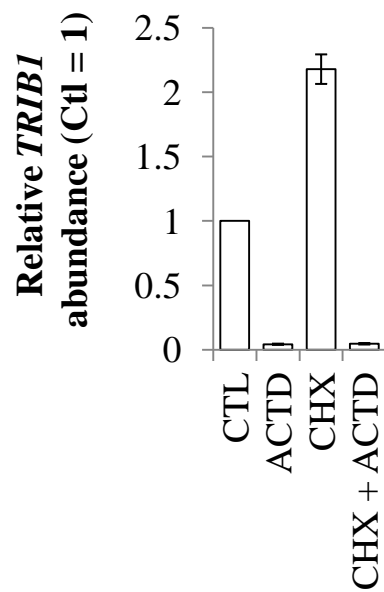

Supplement: S1 Fig — TRIB1 transcript was measured in HeLa cells in response to CHX and/or ACTD by qRT-PCR. Cells were pre-treated with ACTD for 10 min prior to CHX addition, followed by a co-incubation for 5 h. All changes are statistically different from control (CTL). Inclusion of ACTD prevented the CHX-induced TRIB1 upregulation (4.3 vs 4.6%, p = 0.43). Biological replicates are distinct from those in Fig 2 and represent the average of 3 experiments. Error bars represent S.D. (PDF) [file pone.0152346.s001.pdf]

S2 Fig.

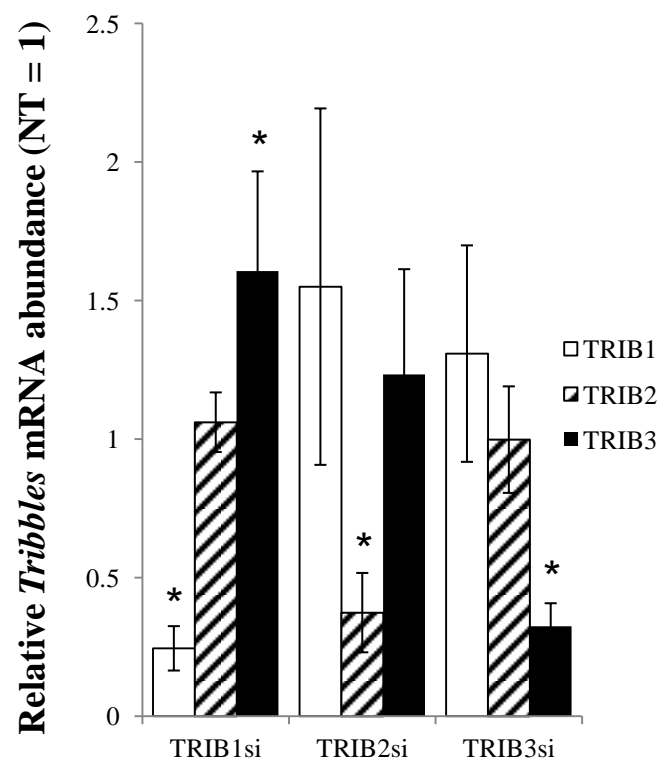

Supplement: S2 Fig — HepG2 cells were transfected with the indicated tribbles specific siRNA for 48 h and the levels of the tribbles mRNA quantified by qRT-PCR. Results represent the mean of 4 experiments (± 95% C.I).* indicates statistical significance (p < 0.05, Student’s unpaired, 2-tailed, t-test). (PDF) [file pone.0152346.s002.pdf]

S3 Fig.

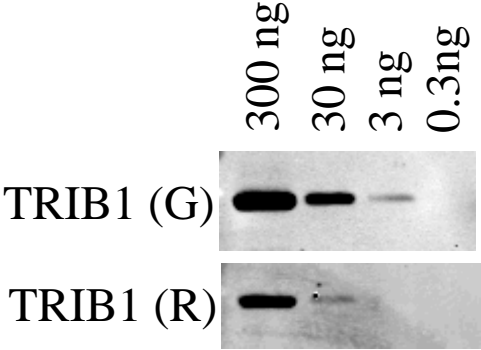

Supplement: S3 Fig — Purified GST TRIB1 fusion proteins were isolated from BL21 bacteria and quantified relative to bovine serum albumin. Glutathione eluted proteins were diluted serially, resolved by SDS-PAGE and transferred to nitrocellulose. Western blot was performed using 0.5 ug of either rabbit or goat TRIB1-specific antibody and cognate secondaries (LI-COR; Donkey anti rabbit 680 and Donkey anti goat 800). (PDF) [file pone.0152346.s003.pdf]

S4 Fig.

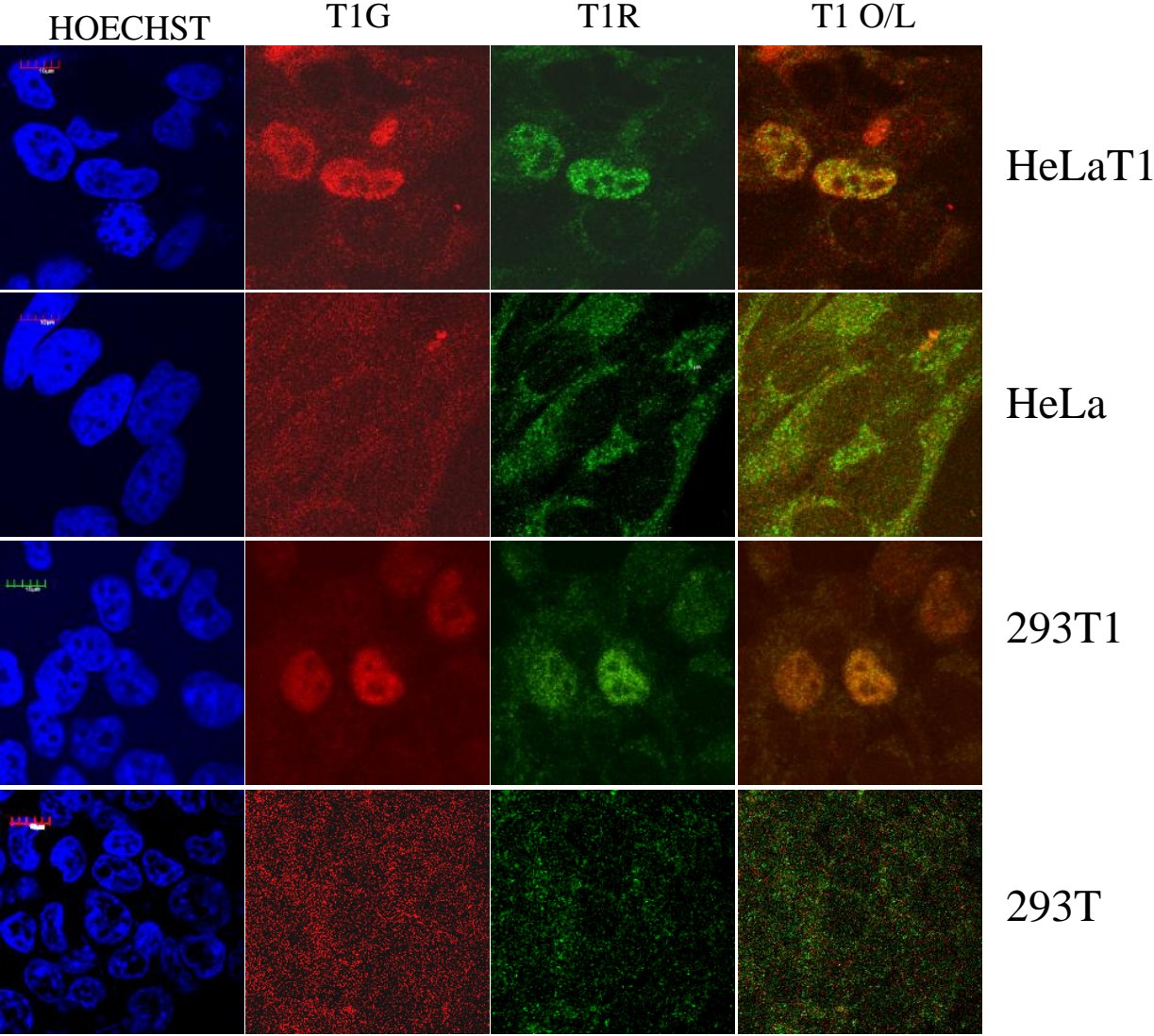

Supplement: S4 Fig — HeLa and 293T cells were stably transduced with lentiviral particles encoding for TRIB1. Cells were fixed, permeabilized and blocked in 10% horse serum/ PBS. Microscopy was performed using either a donkey antibody targeted towards the C-terminus of TRIB1 (T1G) or the rabbit specific for an epitope contained within AA104-372 (T1R). Overlay of the 2 TRIB1 signals is shown on the right. Scale bar length corresponds to 10 μm. (PDF) [file pone.0152346.s004.pdf]

S5 Fig.

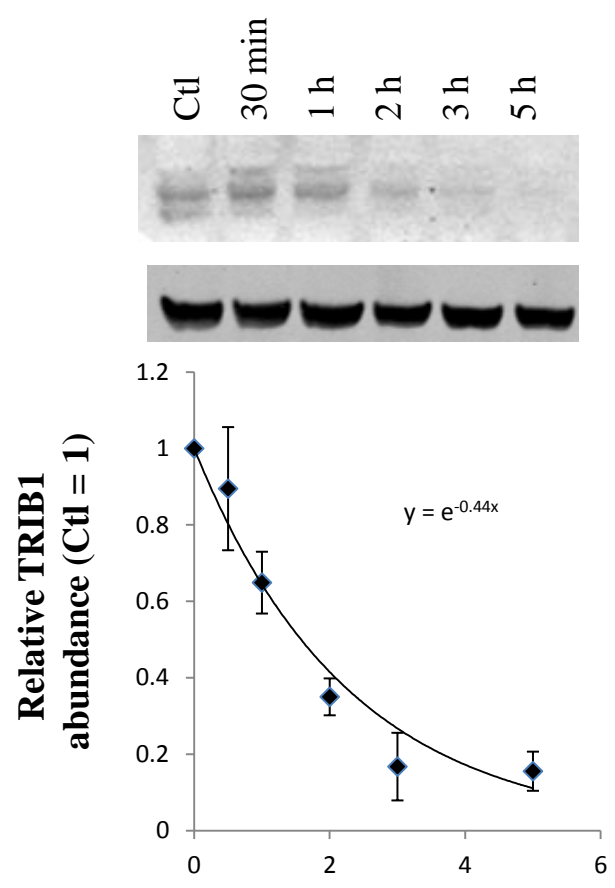

Supplement: S5 Fig — HeLaT1 cells were treated with ACTD (5 μg/ml) for the indicated time. Graphical representation representing the average of 2 experiments (± S.D.) fit to an exponential regression is shown at the bottom; half-life is estimated at 94 min (PDF) [file pone.0152346.s005.pdf]
