# Supplementary material for: TRIB1 Is Regulated Post-Transcriptionally by Proteasomal and Non-Proteasomal Pathways
Source: PLoS One. 2016 Mar 28;11(3):e0152346. doi: 10.1371/journal.pone.0152346 (PMC4809572; doi:10.1371/journal.pone.0152346)
Supplement: S1 Supplementary Materials — (DOC) [file pone.0152346.s006.doc]

**Mutagenesis**

Oligonucleotide pairs used to generate CFPTRIB1 deletion constructs; deletion introduces an EcoRV (GATATC; DI residues) to facilitate screening.

ECFPC1 1-162

ATCCTTCTCAAAGAAGACATAGGCC

ATCTAAGGATCCACCGGATCTAGATAACTG

ECFPC1 162-372

ATCATGGATCCCGGGCCC

ATCAAGGACTTTGGGGACATGC

ECFPC1 92-372

ATCCATGGATCCCGGGCCC

ATCGCCGACTACCTGCTGCTG

ECFPC1 52-372

ATCCATGGATCCCGGGCCC

ATCGAGTGCTCCAGCCCCCC

ECFPC1 32-372

ATCCATGGATCCCGGGCCC`

ATCAAACGCCTGCTGGACGCC

Oligonucleotide pairs used to generate FLAGTRIB1 deletion constructs; deletion introduces an EcoRV (GATATC; DI residues) to facilitate screening. To generate Δ31-51PKIANES, an NES derived from PKIA was inserted in the ECORV site generated as a result of the 31-51 deletion.

PCMVFLAGTRIB1Δ31-51

ATCCGGGACGCCTCGGGTGGC

ATCGAGTGCTCCAGCCCCCC

PCMVFLAGTRIB1Δ31-51PKIANES

TTAGCCTTGAAATTAGCAGGTCTAGAC

GTCTAGACCTGCTAATTTCAAGGCTAA

qRT-PCR oligonucleotides

TRIB1

Total TRIB1

TRIB1F

TTCAAGCAGATTGTCTCCG

TRIB1R

CATCCACACTGGACGCGAG

Endogenous TRIB1

TRIB1F

TTCAAGCAGATTGTCTCCG

TRIB1UTR3rc2

ATGGAAATGCCAGGTGTTAAGAATTA

Exogenous TRIB1

Forward oligonucleotide detects vector specific sequences

PLVXforseq

GTCAGATCGCCTGGAGACG

CGTCCAGCAGGCGTTTG

TRIB2

TGCGTTTCTTGTATCGGGAAATAC

CATAGCTTCGCTCAAAGAACAC

TRIB3

CTGCCCTACAGGCACTGAGT

AGCACGGCAGCCTCAGGCTCA

siRNAs:

siRNA were obtained from Life Technologies (Ambion).:

Negative control #1 (NT#1)

TRCPB s17110

CUL1 s16054

TRIB1 siRNA (Silencer Select) UCUGUUGGGAUGAGUGACU

TRIB2 s26280

TRIB3 s33710

TRIB1-targeting and control ASOs:

ASO1: CAGTTCTGCATTCCTGAGCA (Exon 3)

Non-target ASO (NT): GGAGGAACCTTCAGGGAAGG

Antibodies

Antibodies from GeneTex, Inc

TRIB1g: GTX88755

TUBB: GTX11307

TRIB1r: GTX111960

FLAG: M2 (Sigma)
